# Swimming and rafting of *E.coli* microcolonies at air–liquid interfaces

**DOI:** 10.1002/mbo3.532

**Published:** 2017-10-22

**Authors:** Giorgia Sinibaldi, Valerio Iebba, Mauro Chinappi

**Affiliations:** ^1^ Department of Mechanical and Aerospace Engineering Sapienza University of Rome Rome Italy; ^2^ Public Health and Infectious Diseases Dept Istituto Pasteur Cenci Bolognetti Foundation Sapienza University of Rome Rome Italy; ^3^ Center for Life Nano Science Istituto Italiano di Tecnologia Rome Italy; ^4^ Department of Industrial Engineering University of Rome Tor Vergata Rome Italy

**Keywords:** biofilms, *E.coli*, microcolony, modeling, motility

## Abstract

The dynamics of swimming microorganisms is strongly affected by solid‐liquid and air‐liquid interfaces. In this paper, we characterize the motion of both single bacteria and microcolonies at an air‐liquid interface. Both of them follow circular trajectories. Single bacteria preferentially show a counter‐clockwise motion, in agreement with previous experimental and theoretical findings. Instead, no preferential rotation direction is observed for microcolonies suggesting that their motion is due to a different physical mechanism. We propose a simple mechanical model where the microcolonies move like rafts constrained to the air‐liquid interface. Finally, we observed that the microcolony growth is due to the aggregation of colliding single‐swimmers, suggesting that the microcolony formation resembles a condensation process where the first nucleus originates by the collision between two single‐swimmers. Implications of microcolony splitting and aggregation on biofilm growth and dispersion at air‐liquid interface are discussed.

## 1 INTRODUCTION

Bacteria live in different environments, continually exposed to various stimuli such as chemical compounds and physical constraints. The same bacterial species may express a differential set of genes and a different behavior if surrounding physicochemical conditions change, like gravity (Arunasri et al., 2013; Rosenzweig, Ahmed, Eunson, & Chopra, 2014; Tucker et al., 2007), shear stress (Aprikian et al., 2011; Dingemans et al., 2016; Nickerson, Ott, Wilson, Ramamurthy, & Pierson, 2004), and quorum sensing/quenching (Grandclément, Tannières, Moréra, Dessaux, & Faure, 2015; Tiaden, Spirig, & Hilbi, 2010). In particular, bacterial population size, which quorum sensing depends on, leads to a different motility behavior for single cells or bacterial aggregates, like early‐stage biofilms, known as micro/macrocolonies (Serra & Hengge, 2014; Sutherland, 2001; Teschler et al., 2015). In literature there are several evidences of an inverse correlation among motility and biofilm formation (Caiazza, Merritt, Brothers, & O'Toole, 2007; Guttenplan & Kearns, 2013; Pesavento et al., 2008;), where bacteria stop swimming, adhere to a surface, and start producing an extracellular matrix composed by proteins, exopolysaccharides, DNA, and other species‐specific molecules (Hobley, Harkins, MacPhee, & Stanley‐Wall, 2015; Teschler et al., 2015). In this proposition, it was found that biofilms could be formed at both solid–liquid and air–liquid interfaces in a bacterial broth culture, depending on involved species and their swimming/aerobic properties (Armitano, Méjean, & Jourlin‐Castelli, 2014; Hollenbeck et al., 2014; Spiers, Bohannon, Gehrig, & Rainey, 2003). Even if several models were proposed to explain such a phenomenon (Ardré, Henry, Douarche, & Plapp, 2015; Armitano et al., 2014; Steenackers, Parijs, Foster, & Vanderleyden, 2016; de Wouters, Jans, Niederberger, Fischer, & Rühs, 2015), a proper rheological/microfluidic description of bacterial motility and microcolony formation at air–liquid interface is lacking. Microcolony and biofilm formation at air–liquid interface is of clinical importance, especially in human diseases involving bacterial infections of lungs, such as cystic fibrosis, chronic obstructive pulmonary disease (COPD), primary ciliary dyskinesia (PCD), and asthma (Beck, Young, & Huffnagle, 2012; Livraghi & Randell, 2007).

The swimming of single bacteria and the collective motion of microorganisms have attracted the interest of a varied community. Accumulation at interface (both solid–liquid and air–liquid) was studied with a number of theoretical (Ishimoto & Gaffney, 2013), computational (Costanzo, Di Leonardo, Ruocco, & Angelani, 2012; Mathijssen, Doostmohammadi, Yeomans, and Shendruk, 2016; Theers, Westphal, Gompper, & Winkler, 2016), and experimental approaches (Wioland, Lushi, & Goldstein, 2016), and several puzzling phenomena such as upstream flowing (Mathijssen, Shendruk, Doostmohammadi, Yeomans 2016) and oscillatory motion in microchannel (de Graaf et al., 2016) emerged when bacteria swim under strong confinement. The interaction of flagellated microswimmers with structured surfaces often results in swimmer trapping as shown in Sipos, Nagy, Di Leonardo, & Galajda (2015) for convex wall and in Gu et al. (2016) for grooved surfaces. For a recent review on both single swimmers and collective motion, see Elgeti, Winkler, & Gompper (2015).

Here, we kept *Escherichia coli* as a bacterial model to depict the transition from single swimmer to microcolony motion at air–liquid interface. Single flagellated microswimmers, such as *E. coli*, are attracted by both solid–liquid and air–liquid interfaces (Lopez & Lauga, 2014; Morse, Huang, Li, Maxey, & Tang, 2013). In both cases, circular trajectories are observed, although the direction of rotation is different: at solid–liquid interface the flagellated bacteria swim clockwise (CW) (Lauga, DiLuzio, Whitesides, & Stone, 2006), while counterclockwise swimming (CCW) is observed at air–liquid interface (Di Leonardo et al., 2011; Lemelle, Palierne, Chatre, & Place, 2010). These experimental findings are supported also by fully resolved hydrodynamic simulations of a single flagellated swimmer (Pimponi, Chinappi, Gualtieri, & Casciola, 2016; Shum, Gaffney, & Smith, 2010). The same approach was also employed to single swimming motion in confined geometries (Shum & Gaffney, 2015).

Here, we discuss the motion of single *E. coli* and microcolonies at air‐liquid interface. Our experimental data show that single swimmers and microcolonies coexist at air–liquid interface. Although both of them follow circular trajectories, single bacteria preferentially show a counterclockwise motion, while no preferential rotation direction is observed for microcolonies. Microcolonies move like rafts constrained to the air–liquid interface. A simple physical model is proposed to explain their motion. In addition, our data show that collisions between microcolonies or between single‐swimmers and microcolonies often result in a merging and that, occasionally, a small colony detaches from a large colony and starts an independent rafting.

## 2 MATERIALS AND METHODS

### 2.1 Preparation of *E. coli* cell suspension

A single colony of *E. coli* MG1655 strain (DSM #18039) was picked up from a MacConkey Agar No.3 plate (cat# CM0115, Oxoid), and grown overnight at C, 265 g, in 1 ml of Tryptone Broth (TB) containing 1% wt/vol Bacto Tryptone (Bacto Tryptone, cat# 211705, BD Biosciences) and 0.8% wt/vol NaCl. The saturated culture was then diluted 1:100 into fresh medium (1 ml TB) and grown for 3.5 hr, 265 g, at until reaching mid‐log phase (OD600 = 0.5). Bacterial cells were harvested from culture media by centrifugation (2.200 g, 10 min) at room temperature, and the pellet was resuspended by gently mixing, avoiding pipetting, in prewarmed motility buffer [10 mmol/L potassium phosphate, 0.1 mmol/L Na‐EDTA (pH 7.0), 76 mmol/L NaCl, and 0.002% Tween 20]. This process was repeated three times to achieve growth medium depletion and a suitable final bacteria concentration (Min et al., 2009).

### 2.2 *E. coli* visualization with cavity slide

**Figure 1:**
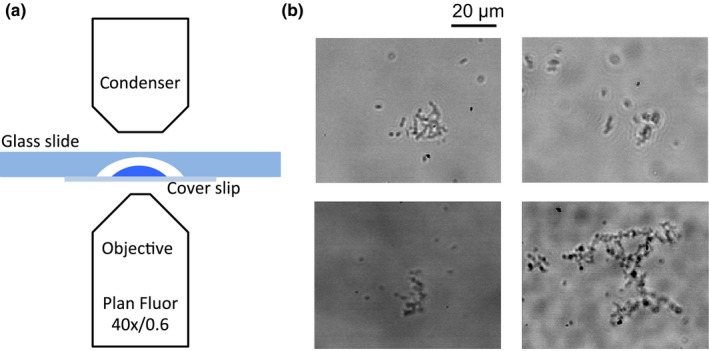
(a) Sketch of the experimental set‐up. The *E.coli* suspension is dropped onto the coverslip stuck on the cavity slide. The cavity slide is kept upside‐down in the inverted microscope slide holder. (b) Typical snapshots. Single *E.coli* and microcolonies coexist. Different microcolonies configuration can be observed

Two microliters of *E. coli* suspension were dropped onto a 22‐mm squared borosilicate coverslip (cat# 12‐553‐454, Fisher Scientific), and this latter was stuck on a cavity slide (cat# S99369, Fisher Scientific) using distilled water. The hanging drop of *E. coli* suspension was kept upside‐down (“reversed hanging drop”), with coverslip in direct contact with the microscope objective, in order to minimize the gravity‐driven concentration of bacteria on the air–liquid interface at the top area (Di Leonardo et al., 2010). A sketch of this configuration is shown in Figure 1a.

### 2.3 Image acquisition

The images were acquired by means of a Photron miniUX100 fast camera connected to an inverted microscope Zeiss Observed Z1. The acquisitions were made at a frame rate of 50 fps using a LD Plan Neofluar 40X/0.6 NA Zeiss objective. Image acquisition set‐up is sketched in Figure 1a. Typical snapshots are reported in Figure 1b.

### 2.4 Trajectory analysis

Single *E. coli* movements were tracked using the Mosaic plugin (Sbalzarini & Koumoutsakos, 2005) for Image‐J (Abràmoff, Magalhães, & Ram, 2004). Only trajectories longer than 70 frames (1.4 *s*) were considered. The final output was then manually filtered to remove bacteria that did not show a coherent motion and the bacteria belonging to microcolonies. Average velocities were calculated with an in‐house code while the radius of curvature was determined via least square fitting adapting the python code available at http://www.scipy.org/Cookbook/Least_Squares_Circle (Jones et al., 2001). Concerning the microcolonies, at the first frame we identified the center of the microcolony and selected two bacteria belonging to the microcolony and quite far from its center. Then we tracked the position of these two bacteria using the MtrackJ (Meijering et al., 2012) plugin for imageJ. Angular velocity and trajectory of the raft center were then calculated by using the standard kinematic relation for 2D rigid bodies. Average radius of curvature of microcolony center was determined as for single swimmers, trajectory with *R *> 50 μ*m* (∼10% of the cases) was discarded as they correspond to trajectories where a univocal direction of rotation is not apparent or where the different methods of least square fitting did not provide coherent estimation of *R*.

## 3 RESULTS

### 3.1 Single *E. coli* swimmer

**Figure 2:**
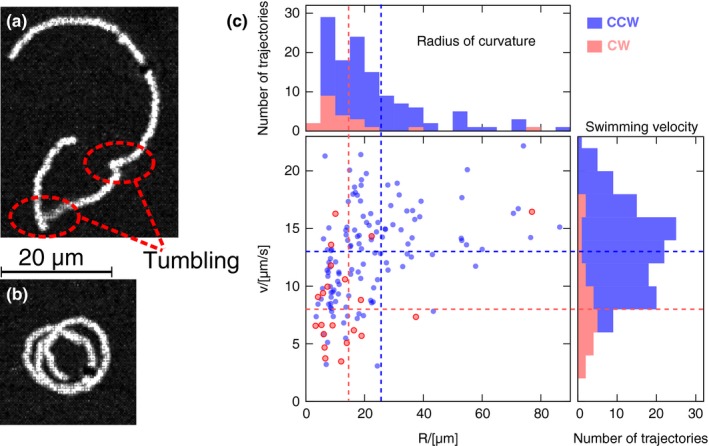
Single *E.coli* swimmers. The microswimmer trajectories are constituted by a sequence of circular arcs (a,b). The cusps between two consecutive arcs correspond to tumbling phases. In few cases, complete circles are apparent. C) Radius of curvature *R* versus swimming velocity *v*. Each point corresponds to a single circular arc. Red and blue points refer to CW and CCW trajectories, respectively. Horizontal and vertical dashed lines are the mean values. CCW swimmers move significantly faster than CW swimmers (*p* < 10^−6^) while radius of curvature difference is not statistically significant (*p* > .05)

In all the analyzed image sequences, the single bacteria swim in circular trajectories. In few cases, complete circles are apparent (Figure 2b), while the more frequent condition is characterized by circular arcs possibly connected by cusps (Figure 2a). Each cusp corresponds to a tumbling phase where the *E. coli* momentarily stops its motion to change swimming direction. Single bacteria preferentially swim counterclockwise (CCW), with only 14% of the them swimming clockwise (CW).

For each trajectory, we calculated the average radius of curvature *R* and the average swimming speed *v*. The distribution of *R* and *v* is reported in Figure 2c for both CCW (blue) and CW (red). CW swimmers are slower than CCW ones (*p* < 10^−6^), while no statistically significant difference is found concerning the radius of curvature *R* (*p* > .05).

### 3.2 Microcolonies

As observed for single swimmers, also microcolonies follow curved trajectories. In particular, microcolonies move like rigid rafts trapped at the air–liquid interface. In *E. coli*, the extracellular matrix (EM) is promptly released when bacteria respond to a quorum sensing signal or when facing a physico/chemical stimulus for an optimal niche adaptation: at that point, bacteria change their behavior from motile to EM producers. Microcolony rafts are immediately produced at both solid–liquid and air–liquid interfaces (Armitano et al., 2014), even if we observed a significant higher prevalence of microcolonies at air–liquid rather than solid–liquid. In the present experiment, measurable bacterial rafts formed at air–liquid till the first hour after the preparation of bacterial suspension in accordance with a previously suggested model (Ardré et al., 2015).

**Figure 3:**
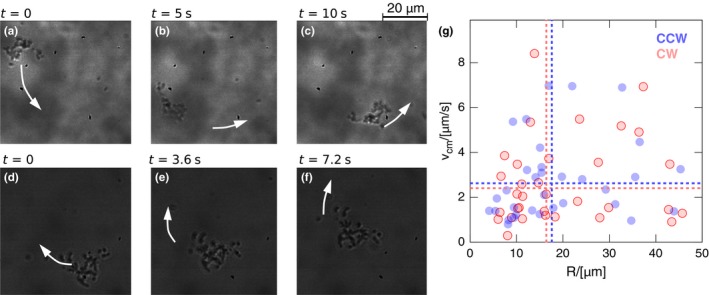
Microcolonies move like 2D rigid rafts suspended at the air–liquid interface and exhibit both CCW (a–c) and CW motion (d–f). Panel G reports the scatter plot of the speed versus the radius of curvature of the microcolony center. Red and blue symbols refer to CW and CCW motion of the raft center, respectively. CW motion occurs 49% of the cases while CCW 51%, the difference is not significant (*p* = .91). Horizontal and vertical lines correspond to the average CW and CCW radius of curvature and speed. No significant difference is observed between CW and CCW for both average speed and radius of curvature *R* (*p* > .2 for both comparisons)

Figure 3 reports snapshots for both CW and CCW motions. The average speed and the radius of curvature *R* of the center of each microcolony are reported in Figure 3g. Several considerations follow. (1) Differently from the single swimmers, microcolonies do not show a preferential direction of rotation. CW and CCW rotations occur with the same probability. (2) The average speed of microcolonies is lower than single swimmer ($\left\langle v_{\mathrm{cm}}\right\rangle =2.31\mu m\;s^{-1}$ for microcolonies, $\left\langle v\right\rangle =12.4\mu m\;s^{-1}$ for single bacteria, *p* < 10^−6^) and no significant difference in the speed of CW and CCW rotating colonies is observed. (3) The radius of curvature does not statistically differ compared to the single swimmer case (〈*R*〉 = 17.68 μ*m* for microcolonies, 〈*R*〉 = 24.4 μ*m* for single bacteria, *p* = .06). The radius of curvature does not show a dependency on the microcolony size. The first two occurrences indicate that the mechanism underlying the microcolony motion is different from the single swimmer. A simplified model is presented in the discussion section.

### 3.3 Microcolony growth and splitting

**Figure 4:**
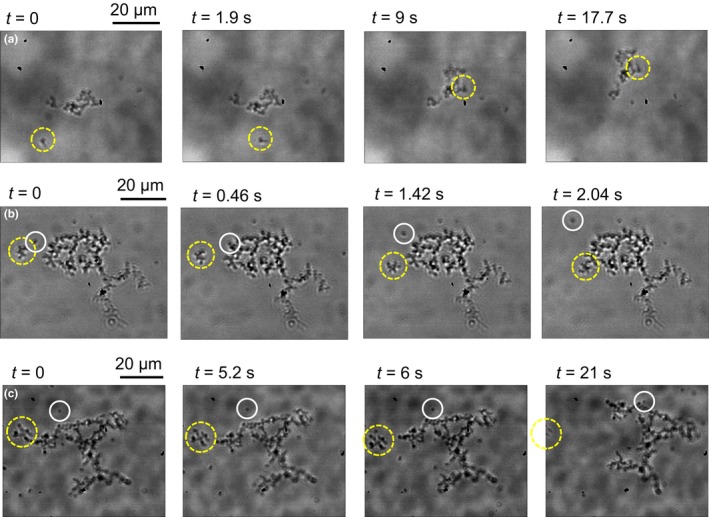
Microcolony growth and splitting. (a) A single swimmer (yellow dashed circle) hits a microcolony and merges with it. (b) A small microcolony (yellow dashed circle) collides with a large raft and merges with it while a single swimmer (white continuous circle) hits the large raft, it is trapped for a while on the raft contour and, finally, escapes. (c) A small portion of a large microcolony (yellow dashed circle) splits off and starts to raft independently while a single bacterium (white continuous circle) first swims at a constant distance form the microcolony and then is trapped by it

Another interesting outcome of our experiments is an insight on the mechanism of the microcolony growth at the air–liquid interface. We observed that the collision of a single bacterium with a microcolony often results in the adhesion of the single swimmer to the microcolony. An example is reported in Figure 4a. The same aggregation mechanism holds also for collisions between microcolonies, see the yellow dashed circle in Figure 4b. Not all the collisions give rise to aggregation, as testified by the trajectory of the single swimmer highlighted by the white continuous circle in Figure 4b. The swimmer hits the raft and it is momentarily trapped at the microcolony border but, after a while, it escapes. A further example is reported in Figure S1 and Video S2, where the collision of single swimmers with a small microcolony results in a partial rearrangement of the bacteria belonging to the raft. The aggregation mechanism based on collision and merging resembles the growth of a liquid droplet in a supersaturated vapor phase, where a single molecule colliding with an already formed droplet can merge into the droplet or be scattered. However, differently from liquid droplet, where the critical nucleus (i.e., the cluster size where the growth due to aggregation and decay due to evaporation balances) is often quite large, for example, 15−30 molecules for water (Matsubara, Koishi, Ebisuzaki, & Yasuoka, 2007), we observed stable *E. coli* microcolonies formed by very few bacteria (<5) and we never observed spontaneous separation (the analogous of evaporation for a liquid droplet) of a single swimmer from the microcolony. This is probably due to the strong adhesion among raft members provided by the extracellular matrix. Instead, we observed microcolony splitting, where a small raft separates from a large microcolony and starts moving independently, see the yellow dashed circle in Figure 4c. This process can potentially accelerate the microcolony dissemination as the novel independent microcolonies constitute stable nuclei that can increase in size after collision with single swimmers.

## 4 DISCUSSION

Clockwise (CW) and counterclockwise (CCW) motion of single flagellated microswimmers close to an interface can be explained in terms of fluid dynamic interaction between the swimmer and the surface. No‐slip boundary condition at the fluid interface gives rise to CW motion (Frymier, Ford, Berg, & Cummings, 1995; Shum et al., 2010), while swimming close to a free‐slip interface results in CCW trajectories (Lauga et al., 2006; Pimponi et al., 2016). In the latter case, the theoretical explanation relies on the method of images, see Di Leonardo et al., 2011. The swimmer motion is affected by the velocity field generated by its mirror image on the other side of the free‐slip interface. The counter‐rotating image of the *E. coli* head produces a lateral velocity on the actual swimmer head. Such velocity gives rise to a corresponding viscous force in the same direction. The same reasoning applies to the counter‐rotating image flagellum, so that a net CCW torque acts on the microswimmer. This simple explanation was recently confirmed by numerical simulation employing the full solution of the Stokes equation around a flagellated microswimmer (Pimponi et al., 2016).

To the best of our knowledge, the only two experimental evidences of *E. coli* CCW motion at air–liquid interface were reported in Di Leonardo et al. (2011) and Lemelle et al. (2010). It is worth noting that, while in Di Leonardo et al. (2011) all the data refer to CCW motion, in Lemelle et al. (2010) both CW and CCW motion were observed at air–liquid interface, an occurrence reported also for a different flagellated microswimmer *C. crescentus*, see Morse et al. (2013). Our data confirm that both rotation directions are possible, although the preferential one is the CCW as predicted by hydrodynamic arguments. The occurrence of a small percentage of CW swimming bacteria can be ascribed to the presence of molecules in the media that can alter the usual free‐slip behavior of an air–liquid interface resulting in a no‐slip or a partial‐slip condition, as proposed in Morse et al. (2013). We expect that the local presence of high concentration of molecules secerned by the bacteria in specific regions would also result in an increase in the local viscosity, an occurrence that can potentially explain the smaller velocity of the CW swimmer. Our findings, together with the early study of Lemelle et al. (2010), raise questions on the proper model for the liquid–air interface when modeling the fluid dynamics of biofilms, a topic that is recently attracting the interest of a multidisciplinary community due to its potential relevance in biofilm formation (see, e.g., Mathijssen et al. 2016).

### 4.1 Microcolonies

**Figure 5:**
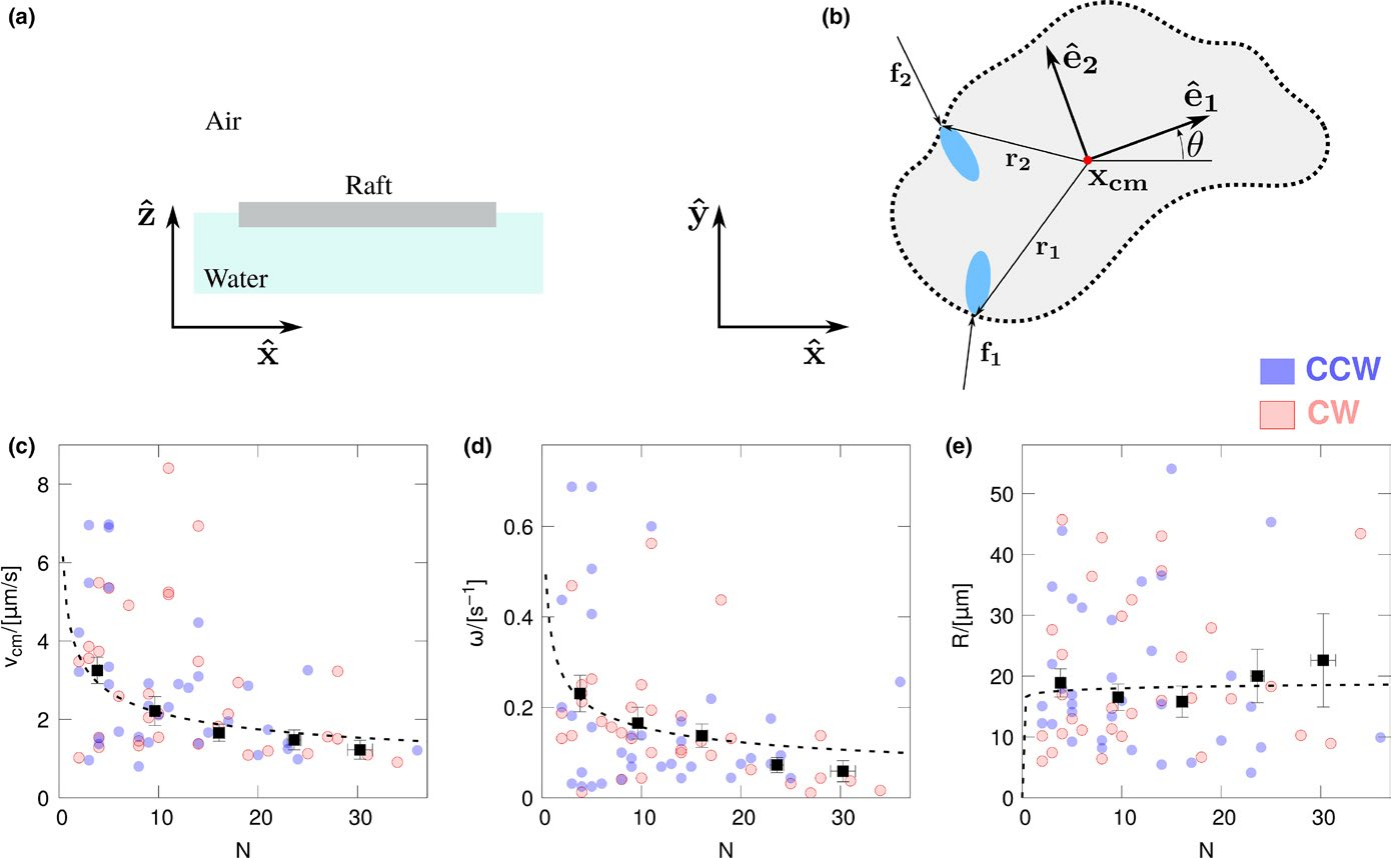
Schematic model of the microcolony. The colony is represented as a raft moving on the air–water interface (panel A). The motion is described by three degrees of freedom, namely, the position of the center of mass **x_cm_ **= (x_cm_, y_cm_) and the angle θ between the unit vector **e**_**1**_ of the body reference frame and the *x*‐axes of the fixed reference frame. The bacteria at the contour of the raft exert a force on the raft, see, for example, **f_1_** and **f_2_** in panel B. Panels C, D, and E report the average velocity of the microcolony center *v*_cm_, the angular velocity of the microcolony ω, and the radius of curvature of the trajectory of the microcolony center as functions of the number of bacteria forming the microcolony (N). Red and blue circles correspond to raw data for CW and CCW rotation of the microcolony center, respectively. Black points represent binned data while dashed lines correspond to power‐law fits

As shown in the results section, also microcolonies follow circular trajectories, however, no preferential rotation direction is observed. Here, we introduce a simple physical model that allows to partially explain this result. In particular, we are able to explain the rotational motion, the absence of a preferential rotation direction, and to propose the scaling law for the microcolony speed *v*_cm_, angular velocity ω, and the radius of curvature *R* with the number of bacteria belonging to the microcolony (*N*). In our toy model, the microcolony moves like a raft constrained at the air–water interface. The raft is kept in motion by the trust exerted by the flagella of the bacteria on its contour, see Figure 5a and b. We exclude the possible contribution of bacteria in the interior of the raft as we expect that, when inside the microcolony, the bacteria change their behavior from motile to EM producers. In addition, their flagella (if present) will point almost perpendicularly to the raft surface and, hence, their contribution to the total force can be neglected. As a first approximation the raft can be modeled as a 2D rigid body with homogeneous density. The raft position in the fixed reference frame with base $\left\{\hat{x},\hat{y}\right\}$ is identified by the coordinates of the mass center of the raft, ***x*_cm_** = *(x*_cm_ *,y*_cm_ *)*. The orientation of the raft is given by the angle θ between the *x*‐axes of the fixed reference system and the unit vector ***e***_***1***_ of the body fixed frame of reference, see Figure 5b. The equations of motion of the raft read

(1) $$m\;{\ddot{x}}_{\mathrm{cm}}=F$$

(2) $$I_{Z}\ddot{\theta }=T$$

where *m* and *I*_*z*_ are the mass and the moment of inertia of the raft, while ***F*** is the total force acting on the raft and *T* is the total torque acting on the raft calculated with respect to an axis parallel to z‐axis and passing through ***x*_cm_**. Only two forces act on the raft: the drag due to the liquid viscosity and the trust exerted by bacteria on its contour.

Concerning the viscous contribution, as the Reynolds number is low, we can safely assume that the drag ***F*_μ_** is proportional to the raft velocity and that the torque *T*_μ_ exerted by the fluid is proportional to the angular velocity $\omega =\dot{\theta }$, in formulae

(3) $$F_{\mu }=-Dv_{\mathrm{cm}}$$

(4) $$T_{\mu }=-G\omega$$

with *D* and *G* constant coefficient depending on the raft shape. For the bacterial trust contribution, we assume that each *E. coli* on the raft contour located at position ***r***_***i***_ in the body fixed reference frame exerts a force ***f***_***i***_ on the raft, see Figure 5b. Hence, the contribution of bacterial trust to total force ***F*** and torque *T* is given by

(5) $$F_{b}=\sum_{i=1}^{N_{b}}f_{i},$$

(6) $$T_{b}=\left(\sum_{i=1}^{N_{b}}r_{i}\times f_{i}\right)\cdot \hat{z}$$

where *N*_*b*_ is the number of bacteria on the raft contour.

Substituting the Equations (3), (4), (5), and (6) into the equations of motion (1) and (2) and neglecting the inertial term, we end up with the following expression for raft velocity and angular velocity:

(7) $$v_{\mathrm{cm}}=\frac{F_{b}}{D}$$

(8) $$\omega =\frac{T_{b}}{G}$$

Assuming that the forces ***f*_*i*_** exerted by the *E. coli* on the raft move together with the raft frame of reference, the solution is a uniform rotational motion with radius of curvature:

(9) $$R=\frac{\left|v_{cm}\right|}{\omega }=\frac{\left|F_{b}\right|G}{T_{b}D}$$

A positive *R* corresponds to CCW motion while negative *R* to CW. It is worth noting that the sign of *R* depends only on *T*_*b*_. If the orientation and the distribution of ***f*_*i*_** are unbiased, for each microcolony positive and negative *T*_*b*_ have the same probability and, consequently, CW or CCW motion occur with the same frequency. Hence, this simple model easily explains the main observations of our work, that are, the circular motion of the microcolony and the absence of a preferential direction in the microcolony rotation.

The model can be further exploited to try to predict the dependency of *v*_cm_, ω, and *R* on the raft size, Figure 5c, d and e. Given Equations (7) and (8), the problem reduces to find reasonable expressions for the scaling of *F*_b_, *T*_b_, *D,* and *G* with the number *N* of bacteria forming the microcolony. Let us start from *F*_*b*_. The *x* and *y* components of the force exerted by a single bacterium at the raft contour are *f*_*i,x*_ *= f cos*α_*i*_ and *f*_*i,y*_ *= f sin*α_*i*_, with α_*i*_ the angle between ***f*_*i*_** and the fixed reference frame axis ***e*_*1*_** and *f* the force intensity, assumed to be the same for all the bacteria. As a first approximation, we consider α_*i*_ as independent, identically, and uniformly distributed random variables. In the limit of large *N*_*b*_, the central limit theorem implies that the *x* and *y* components of the total force *F*_*b*_, dubbed as *F*_*x*_ and *F*_*y*_, follow a Gaussian distribution centered in zero and with standard deviation:

(10) $$\sigma_{F_{x}}=\sigma_{F_{y}}=\frac{1}{\sqrt{2}}fN_{b}^{0.5}$$

Hence, the typical intensity of the total force for a single microcolony scales as:

(11) $$F_{b}∼fN_{b}^{0.5}$$

In the supporting information, we show that Equation (10) can be derived also from standard results on the sum of independent and identically distributed random variables. Equation (10) is hence valid for any *N*_*b*_. In addition, in supporting information, we also provide further details on the calculation of the numerical prefactor in Equation (10). However, it is worth noting that, in the following, the exact value of the prefactor is not relevant as, in our scaling arguments, we will employ only Equation (7).

The scaling of the drag coefficients *D* is less trivial. Standard Stokes flow solutions for oblate ellipsoids suggest that *D*~ *L*, where *L* is the characteristic size of the microcolony. As *N*_*b*_ scales as *L*, we get the following approximate scaling:

(12) $$D∼N_{b}$$

Taken together, Equations (7) and (8), substituted into (7), lead to:

(13) $$v_{cm}∼N_{b}^{-0.5}∼N^{-0.25}$$

with *N* the number of bacteria belonging to the microcolony.

Fitting the raw data on the power law $v_{cm}=a_{v}N^{b_{v}}$ gives *b*_v_ = −0.31 ± 0.08 (dashed line in Figure 5c), in agreement with the model prediction *b*_v_ = −0.25.

A similar argument can be worked out for the raft rotation. Each single bacterium contributes to the total torque *T*_*b*_ with a torque *t*_*i*_ = |***r*_i_**| |***f*_i_**| *sin*φ_*i*_, where φ_*i*_ is the angle between the vectors ***r*_*i*_** and ***f*_*i*_**. Assuming for simplicity that |***r*_i_**| = *r* and |***f*_i_**| = f for all the bacteria, that is, that the microcolony is circular and that the intensity of the force exerted by each bacterium is the same, we have

(14) $$T_{b}=rf\sum_{i=1}^{N_{b}}sin\varphi_{i}$$

We can employ the statistical arguments already used to derive Equations (10) and (11) to deduce that *T*_*b*_ distribution has zero mean and standard deviation given by

(15) $$\sigma_{T_{b}}=\frac{1}{\sqrt{2}}rfN_{b}^{0.5}$$

The number of bacteria at the microcolony contour scales as the microcolony radius *r*, hence $T_{b}∼fN_{b}^{1.5}$. Using again the Stokes flow solutions for oblate ellipsoids, we have *G~L* ^*3*^, and consequently $G∼N_{b}^{3}$,$\left|\omega \right|∼N_{b}^{-1.5}∼N^{-0.75}$, and $\left|R\right|∼N_{b}∼N^{0.5}$. These predictions do not agree with the data. In particular, indicated with $b_{\omega }$ and $b_{R}$ the scaling exponents obtained from the data fitting for ω and *R*, respectively, we have *b*_ω_ = −0.36 ± 0.14 and *b*_R_ = −0.025 ± 0.010 (dashed lines in Figure 5d and e). These discrepancies indicate that our simple model is not able to completely catch the complex physics ruling the dynamics of active particles at air–liquid interfaces. The hydrodynamics of active and passive particles trapped at the interface between two immiscible fluids is a topic that has been attracting the interest of a wide community (Boniello et al., 2015; Dani, Keiser, Yeganeh, & Maldarelli, 2015; Koplik & Maldarelli, 2017; Malgaretti, Popescu, & Dietrich, 2016) and a detailed discussion is out of the aim of this study. Keeping our argumentation in the framework of the presented toy model, our results indicated that we are slightly underestimating the torque or overestimating the drag (or both of them). We do not have data to strongly support one hypothesis with respect to the other. Nevertheless, we would like to briefly present few arguments as stimulus for further work. Concerning the rotational drag *G* at air–liquid interface, an additional contribution associated with the triple‐line fluctuation on the microcolony boundary can potentially overwhelm the standard viscous drag (Boniello et al., 2015). However, also this contribution should scale as *L* ^*3*^, hence, we suggest that the main source of error in the toy model is not due to an overestimation of the rotational drag but to an underestimation of the torque due to the bacteria. Indeed, several mechanisms can lead to a larger torque than the one employed in our toy model. For instance, *E. coli* located in the bulk of the raft can point their flagella (if present) only toward the liquid phase and perpendicularly to the liquid–air interface. The flagella rotation can, hence, increase the torque *T*_*b*_ acting on the raft. The presence of these additional torque sources will not affect the results obtained for velocity scaling, Equation (13), as the force *F*_*b*_ is not altered by contributions perpendicular to the liquid–air interface. Anyway, our results do not allow to completely clarify this issue.

To summarize, we reported experimental data on *E. coli* motion at air–liquid interface. We characterized the motion of both single swimmers and microcolonies. Circular trajectories were observed in both cases. Single swimmers (flagellated bacteria) preferentially swim counter‐clockwise, while microcolonies show no preferential swimming direction. The single‐swimming motion is explained via well‐established theoretical and numerical models (Di Leonardo et al., 2010; Pimponi et al., 2016). For microcolonies motion, we proposed a simple mechanical model where the colony is described as a raft suspended at the air–liquid interface and each bacterial cell at the raft contour exerts a trust. This toy model allows to qualitatively explain why no preferential rotation direction exists and to predict the scaling of raft velocity, angular velocity on the raft size. Only the scaling for raft velocity agrees with the data, suggesting that the approximation made on the raft rotational drag and/or on the torque exerted by the bacteria were too crude to catch the complex physics of active particles at air–liquid interface. As a last conclusion, we reported evidences on aggregation by collision and disgregation phenomena of pre‐formed microcolonies. Our data suggest that collision is an important mechanism for microcolony growth, and it could have pitfalls in clinics. In lungs of healthy people, the movement of cilia usually removes efficiently the periciliar mucus eventually embedding large microcolonies rafts, while in lung diseases, such as cystic fibrosis, this phenomenon is impaired by a thick and viscous mucus layer allowing collision events. We also observed disgregation events where a small portion of large microcolony splits and starts rafting independently. This last phenomenon can potentially play a relevant role in propagation of infections through biofilm dispersal, as seen in microfluidics experiments mimicking blood vessel conditions (Liu et al., 2011). The periciliar fluid of cystic fibrosis subjects has a diminished shear stress (0.5 dyne/cm, Tarran et al., 2005; Iebba et al., 2014) which favors collision events while diminishing microcolonies dispersal, thus our results could explain what should happen in such a scenario. Future directions of the present model would encompass dynamic experiments to simulate different shear‐stress conditions, thus envisioning a broader behavior of microcolonies at air–liquid interface.

## CONFLICT OF INTEREST

We declare that we have no conflict of interest to this work.
