# Supplementary material for: Swimming and rafting of E.coli microcolonies at air–liquid interfaces
Source: Microbiologyopen. 2017 Oct 22;7(1):e00532. doi: 10.1002/mbo3.532 (PMC5822344; doi:10.1002/mbo3.532)
Supplement: Supplementary file 1 [file MBO3-7-na-s001.pdf]

Supplementary information for “Swimming and rafting of E.coli microcolonies at air-liquid interfaces”

Giorgia Sinibaldi, Valerio Iebba, Mauro Chinappi

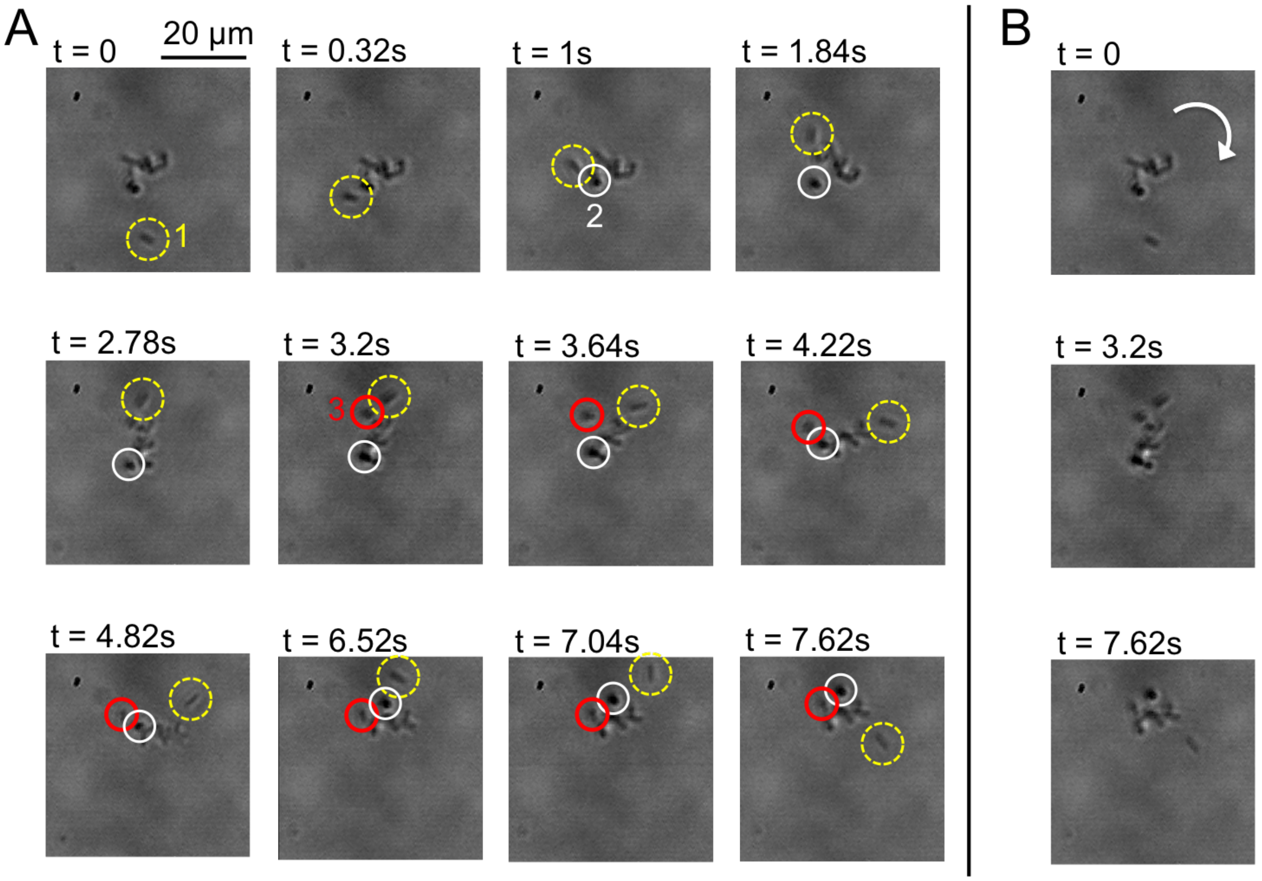

Figure S1. Microcolony interaction with single swimmers at different times. A) Single swimmer #1 (yellow dashed circle) hits the microcolony ($t = 0.32\,s$) and it is trapped starting to rotate with it, and finally it escapes ($t = 4.22\,s$). Single swimmer #2 (white continuous circle) from $t = 1\,s$ and $t = 3.2\,s$ detaches from the microcolony and reattaches at a different location, starting to rotate with the colony. From $t = 4.82\,s$ it detaches again from the microcolony. Single swimmer #3 (red continuous circle) changes its location in the microcolony. At $t = 3.2\,s$ it starts to detach and reattaches at $t = 6.52\,s$ in place of bacterium #2. B) Three snapshots of the microcolony to better understand the change of morphology and direction of rotation.

Video S2. Microcolony interaction with single swimmers. The video refers to same case as Figure S1.

Section S3: Demonstration of equation (10)

Equation (10) of the paper can be obtained with standard arguments here reported for reader convenience. As a first step, we recall that the mean value of the sum of independent and identically distributed (i.i.d.) random variables is equal to the sum of the means of the single variables. The same occours for the variance[1]. Let us apply this standard result to our case. Indicated as F_x the x-component of $\mathbf{F_b}$, from equation (5) of the paper, we have

$$F_x = \sum_{i=1}^{N_b} f \cos(\alpha_i) \ , \tag{1}$$

where, as in the paper, α_i is the angle between $\mathbf{f_i}$ and the fixed reference frame axis $\mathbf{e_i}$ and $f = \mathbf{f_i}$ is the force intensity, assumed to be the same for all the bacteria. If α_is are i.i.d. variables, the mean value of F_x is given by

$$\langle F_x \rangle = \sum_{i=1}^{N_b} f \langle \cos(\alpha_i) \rangle \tag{2}$$

where $\langle \cdot \rangle$ indicates the ensemble average. Since α_i is uniformly distributed in the interval $[0, 2\pi]$, we have $\langle \cos(\alpha_i) \rangle = 0$ and, consequently, $\langle F_x \rangle = 0$. We can repeat the same calculation for the variance

$$\sigma_{F_x}^2 = \langle F_x^2 \rangle = \sum_{i=1}^{N_b} f^2 \langle \cos^2(\alpha_i) \rangle = \frac{1}{2} N_b f^2 \ , \tag{3}$$

where, in the last equation, we used the standard results $\int_0^{2\pi} \cos^2(\alpha) d\alpha = \pi$. The same arguments holds also for F_y and T_b.

[1] See, among others, Ewens, W. J. and Grant G. R. Statistical methods in bioinformatics: an introduction, 2006, Springer Science & Business Media.
